# Skin Involvement by Hematological Neoplasms with Blastic Morphology: Lymphoblastic Lymphoma, Blastoid Variant of Mantle Cell Lymphoma and Differential Diagnoses

**DOI:** 10.3390/cancers15153928

**Published:** 2023-08-02

**Authors:** Magda Zanelli, Francesca Sanguedolce, Maurizio Zizzo, Valentina Fragliasso, Giuseppe Broggi, Andrea Palicelli, Giuseppe Gaetano Loscocco, Camilla Cresta, Cecilia Caprera, Matteo Corsi, Giovanni Martino, Alessandra Bisagni, Marialisa Marchetti, Nektarios Koufopoulos, Paola Parente, Rosario Caltabiano, Stefano Ascani

**Affiliations:** 1 Pathology Unit, Azienda USL-IRCCS di Reggio Emilia, 42123 Reggio Emilia, Italy; andrea.palicelli@ausl.re.it (A.P.); alessandra.bisagni@ausl.re.it (A.B.); marialisa.marchetti@ausl.re.it (M.M.); 2 Pathology Unit, Policlinico Riuniti, University of Foggia, 71122 Foggia, Italy; francesca.sanguedolce@unifg.it; 3 Surgical Oncology Unit, Azienda USL-IRCCS di Reggio Emilia, 42123 Reggio Emilia, Italy; maurizio.zizzo@ausl.re.it; 4 Laboratory of Translational Research, Azienda USL-IRCCS di Reggio Emilia, 42123 Reggio Emilia, Italy; valentina.fragliasso@ausl.re.it; 5 Department of Medical and Surgical Sciences and Advanced Technologies “G.F. Ingrassia” Anatomic Pathology, University of Catania, 95123 Catania, Italy; giuseppe.broggi@phd.unict.it (G.B.); rosario.caltabiano@unict.it (R.C.); 6 Department of Experimental and Clinical Medicine, CRIMM, Center of Research and Innovation of Myeloproliferative Neoplasms, Azienda Ospedaliera-Universitaria Careggi, University of Florence, 50134 Florence, Italy; gloscocco@unifi.it; 7 Doctorate School GenOMec, University of Siena, 53100 Siena, Italy; 8 Pathology Unit, Azienda Ospedaliera Santa Maria di Terni, University of Perugia, 05100 Terni, Italy; cresta.camilla@gmail.com (C.C.); ceciliacaprera@libero.it (C.C.); m.corsi@aospterni.it (M.C.); g.martino1@studenti.unica.it (G.M.); s.ascani@aospterni.it (S.A.); 9 Hematology, Centro di Ricerca Emato-Oncologica—C.R.E.O., University of Perugia, 06129 Perugia, Italy; 10 Second Department of Pathology, Medical School, National and Kapodistrian University of Athens, Attikon University Hospital, 15772 Athens, Greece; koufonektar@yahoo.com; 11 Pathology Unit, Fondazione IRCCS Casa Sollievo della Sofferenza, 71013 San Giovanni Rotondo, Foggia, Italy; p.parente@operapadrepio.it

**Keywords:** lymphoblastic lymphoma, mantle cell lymphoma, blastoid variant, diffuse large B-cell lymphoma, leg type, myeloid sarcoma, multiple myeloma, plasmablastic lymphoma, blastic plasmacytoid dendritic cell neoplasm

## Abstract

**Simple Summary:**

Pathologists working with skin biopsies of different hematological malignancies via blastic morphology need to consider a broad spectrum of diseases that, despite similar blastic features, have different outcomes and require distinct therapeutic strategies. Therefore, a precise and prompt diagnosis is critical for patient management. The aim of our paper is to discuss, in detail, the clinical and pathological features of lymphoblastic lymphoma of B- or T-cell origin and mantle cell lymphoma, in particular its aggressive blastoid variant. Although mantle cell lymphoma rarely involves the skin, its blastoid variant is commonly encountered in cutaneous infiltrates, possibly due to the increased tendency of aggressive subtypes for skin involvement. When aggressive lymphomas are detected at cutaneous sites, a complete systemic work-up is critical. Besides blastoid mantle cell lymphoma and lymphoblastic lymphoma, other hematological malignancies with blastic characteristics and skin involvement are discussed.

**Abstract:**

Hematological neoplasms sharing a blastic morphology may involve the skin. The skin may be either the primary site of occurrence of hematological malignancies with blastic features or cutaneous lesions are the first manifestation of an underlying systemic malignancy. The assessment of skin biopsies of hematological neoplasms with blastic features poses diagnostic problems and requires expert hematopathologists considering a wide range of differential diagnoses. The precise diagnosis of diseases sharing blastic features but with different outcomes and requiring distinct therapies is essential for patient management. The present paper mainly focuses on cutaneous involvement of the blastoid variant of mantle cell lymphoma and lymphoblastic lymphoma of B-cell or T-cell origin. The relevant literature has been reviewed and the clinical aspects, pathological features, prognosis, and therapy of both blastoid mantle cell lymphoma and lymphoblastic lymphoma involving the skin are discussed. A focus on other hematological entities with blastic features, which may involve the skin, to be taken into consideration in differential diagnosis is also given.

## 1. Introduction

The skin may be either secondarily involved in systemic hematological malignancies or represent the primary site of occurrence of a hematological disease. The evaluation of skin biopsies involved in hematological malignancies with a blastic morphology is a diagnostic challenge necessarily leading pathologists to consider a rather broad spectrum of differential diagnoses. Therefore, an accurate diagnosis is crucial for appropriate patient management.

This report aims to provide insights into the cutaneous manifestations of neoplasms with a blastic morphology, focusing on the blastoid variant of mantle cell lymphoma (MCL) [1,2,3,4,5,6,7,8,9,10,11,12,13,14,15,16,17,18,19,20,21,22,23,23,23,24,25,26,27,28,29,30,31,31,32,33,34,35,36,37,38,39,40,41,42], T-cell acute lymphoblastic leukemia/lymphoma (T-ALL/LBL), and B-cell acute lymphoblastic leukemia/lymphoma (B-ALL/LBL) [43,44,45,46,47,48,49,50,51,52,53,53,54,54,55,56,57,58,58,59,60,61,62,63,64,65,66,67,68,69,70,71,72,73,74,75,76,77,78,79,80], through an accurate review of the pertinent literature.

Other diseases with blastic morphology, which may involve the skin, are also discussed. 

An accurate diagnosis of these diseases requires expert hematopathologists. Blastoid MCL cells morphologically resemble lymphoblasts of LBL and in the diagnostic work-up of lymphoid neoplasms with blastic features, the immunohistochemical panel should include cyclin D1 and markers of lymphoid immaturity such as TdT as diagnostic hallmarks of MCL and LBL, respectively. Blastoid MCL may also be difficult to recognize because it can show an aberrant immunophenotyping (negativity for CD5 and positivity for CD23 and CD10). 

Less than 7% of all cutaneous lymphomas originate from lymphoid precursors and the majority of cases are represented by B-LBL, which may involve the skin in 15–33% of cases as a secondary manifestation of a systemic disease or presenting as exclusively cutaneous lesions [48,49,50,51,52,53,54,55,56,57,58,59,60,61,62,63,64,65,66,67,68,69,70,71,72,73]. Cutaneous manifestations in T-LBL are very rare compared with B-LBL. Clinicians and pathologists should be aware of both clinical (often rapidly growing reddish nodule on the head and neck area in young individuals) and pathological features of cutaneous LBL for a prompt diagnosis, starting therapy without delay after a complete work-up including immunophenotyping, molecular and cytogenetic analyses, and imaging. 

## 2. Mantle Cell Lymphoma (MCL): General Features

MCL is a mature B-cell lymphoma derived from the mantle zone of lymphoid follicles, accounting for 3% to 10% of non-Hodgkin lymphomas (NHLs) in Western countries [1,2]. The disease occurs in the late sixties with a male to female ratio of about 3–4:1. MCL is typically characterized by translocation t(11;14)(q13;q32) resulting in overexpression of cyclin D1 [1,2]. The disease appears rather heterogeneous in terms of prognosis, with the classical form of MCL characterized by lymph nodes and extranodal sites involvement having a worse behavior compared with the non-nodal leukemic form, characterized by lymphocytosis and splenomegaly and with bone marrow (BM) and spleen involvement, which shows a more indolent clinical course [1,2].

The sites generally involved in the classical form of MCL are peripheral lymph nodes and extranodal sites, in particular the gastrointestinal tract (GI), Waldeyer ring, spleen, and BM. Other sites including the skin may be rarely involved, especially in advanced stage or relapse [1,2].

## 3. Histology, Cytological Variants, and Immunophenotypic Features of MCL

An MCL diagnosis is primarily made based on a histological assessment of the involved tissues. Most cases show a classical histology usually characterized by a monomorphic proliferation of small to medium-sized lymphocytes (slightly larger than normal lymphocytes), with scarce cytoplasm and slightly irregular nucleus, irregularly dispersed chromatin, and generally inconspicuous nucleolus. In the background, reactive lymphocytes of T-cell origin, scattered epithelioid histiocytes, and hyalinized small blood vessels may be present. MCL may show a nodular; diffuse; or, more uncommonly, mantle-zone growth pattern. Besides the classical MCL, morphological variants have been described as small cell variant resembling B-cell small lymphocytic lymphoma/chronic lymphocytic leukemia (B-SLL/CLL) and marginal zone-like MCL resembling marginal zone lymphoma (MZL). 

Two aggressive variants, blastoid and pleomorphic MCL, are challenging to diagnose [1,3,4]. 

In the pleomorphic subtype, cells are larger than in classical MCL, with prominent nucleolus and pale cytoplasm resembling diffuse large B-cell lymphoma (DLBCL), whereas in the blastoid subtype, cells are more monomorphic, medium-sized, with round/oval nuclei, dispersed chromatin, and inconspicuous nucleolus mimicking lymphoblasts of ALL/LBL. 

Both the pleomorphic and blastoid variants of MCL share a high proliferative index and a poor outcome; however, the current WHO classification and ICC classification suggest keeping separate the two variants. There is usually a tendency toward more aggressive, highly proliferative variants at relapse, although the opposite way around may occur [1,5,6,7].

It is very difficult to differentiate MCL from other small cell lymphoma on the basis of morphological features alone without other ancillary techniques; immunophenotypic analysis is essential to establish the diagnosis of MCL, and the use of fluorescence in situ hybridization (FISH) to detect *CCND1* rearrangement can also be helpful for diagnosis.

MCL immunophenotyping features are rather typical with positivity for B-cell markers (CD20, CD79alpha, and PAX5), CD43, CD5, cyclin D1, and SOX11. CD10 and BCL6 are usually negative but may be expressed especially in aggressive variants [8]. Unlike B-cell SLL/CLL expressing CD23, MCL is usually CD23-negative, and this marker labels only some follicular dendritic cells in the background of MCL [1]. The lack of CD200 by flow cytometry is helpful to differentiate MCL from CLL [9]. CD200 may be expressed in the leukemic, non-nodal variant [9]. MCL classically overexpresses cyclin D1, which is associated with the translocation t(11;14)(q13;q32) leading to *IGH::CCND1* fusion, found in more than 95% of cases [1]. SOX11 is detected in the majority of MCL, with the exception of the leukemic non-nodal form typically lacking SOX11 expression and is helpful to identify CD5- or cyclin D1-negative cases [10,11]. It is worth mentioning that SOX11 is not specific for MCL, being expressed also in LBL, Burkitt lymphoma, and hairy cell leukemia [12]. Less than 5% of MCL cases lack cyclin D1 expression generally due to the lack of *CCND1* rearrangements [13]. Half of cyclin D1-negative cases show rearrangement of *CCND2*, and the others have genetic abnormalities causing the expression of cyclin D2 or cyclin D3 [1,14]. In terms of outcome, there is a significant heterogeneity in MCL patients, with a subset showing a prolonged, indolent course (usually leukemic/non-nodal MCL, SOX11 negative, CD200 positive, and mutated IGHV) and a subset with an aggressive course (blastoid or pleomorphic morphology, high Ki67, MYC positive, *TP53* mutations, and del17p) [15,16]. Widely accepted biomarkers predicting prognosis are cytological features, and Ki67 and TP53 immunostaining. The currently accepted Ki67 cut-off is 30%, and patients with Ki67 equal to or greater than 30% are associated with a worse prognosis [17]; high TP53 expression with positivity in 50% of MCL cells correlates with a poor outcome [18].

## 4. Blastoid MCL: General Features

The blastoid variant of MCL accounts for 10–30% of all MCL cases [19,20]. It often arises de novo and less frequently as a transformation from classical MCL [19,20,21,22]. Its clinical presentation resembles that of classical MCL [3,16]. In the largest series of aggressive MCL histology from the MD Anderson Center by Jain et al., of 183 cases with aggressive histological features, 152 were blastoid, 31 were pleomorphic, and most patients were males (75%) with a median age of 65 [19]. Blastoid MCL often has involvement of extranodal sites (20–72%) including BM (50–80%), skin (72%), lung (30%), GI (20–30%), and central nervous system (5–30%) [20,21,22].

The definition of blastoid MCL is based on morphological features with cells resembling lymphoblasts, whereas the presence of high proliferation index (evaluated by Ki67 index) alone is not considered sufficient for a blastoid MCL diagnosis as classical MCL may also display a high proliferation index. Currently, there is a lack of guidelines on how to report cases showing co-existent classical and blastoid cytology, although some authors suggest reporting the cases with combined cytological features as blastoid subtype [3]. The diagnosis of blastoid MCL is usually made at first presentation, although in some cases the morphological features may change from classical cytology at presentation to blastoid at disease progression or relapse; conversely, rare cases that relapse with a classical MCL cytology, showing a blastoid morphology at first diagnosis, are reported [1,3,19,20,21,22]. Histologically, the growth pattern of the blastoid subtype is usually diffuse and less often nodular; an in situ pattern of blastoid MCL is not reported. 

Blastoid MCL may show some immunophenotypic variations, making difficult its correct diagnosis. The blastoid variant may show the loss of CD5 expression (25–28%) [16,23,24,25] and may have the aberrant expression of CD10, BCL6, and CD23; in particular, BCL6 and CD10 positivity combined with the lack of CD5 may lead to an incorrect diagnosis of DLBCL [26]. These immunophenotypic shifts are usually observed during the transformation from classical to blastoid MCL [21,26]. Ki67 is commonly high in the blastoid subtype. c-MYC protein is expressed at higher levels compared with classical MCL, and *MYC* aberrations are often observed; in a series of patients with *MYC* gene rearrangement, 89% showed a blastoid or pleomorphic morphology [27]. 

In addition to t(11;14), the blastoid variant has increased numbers of specific genetic alterations, and a complex karyotype is often identified in blastoid MCL patients (88%) [21]. Abnormalities of chromosomes 13, 18, and 8 are more frequent in blastoid MCL, whereas abnormalities of chromosomes 13, 17, and 3 are found more commonly in pleomorphic MCL. Deletion of 13q and deletion of 18q have been found in 50% and 27% of blastoid MCL, respectively, compared with 20% and 7% of classic MCL [16,28]. Somatic mutations in the *TP53*, *NOTCH1*, *NOTCH2*, and *NSD2* genes are prevalent in blastoid MCL [16]. The TP53 protein is frequently overexpressed and *TP53* genetic alteration is often associated with the blastoid morphology, although not exclusive of this cytological subtype. Blastoid MCL patients belong to the high-risk category (as well as cases with Ki67% =/> 30%, *TP53* mutations, advanced stage, and high-risk MCL International Prognostic Index). The treatment of blastoid MCL patients represents a huge clinical challenge, and these patients often receive intensive chemoimmunotherapy followed by consolidation with autologous stem cell transplant (ASCT). With the introduction of ibrutinib, acalabrutinib, venetoclax, and anti-CD19 CAR-T therapies, the outcome of patients with blastoid MCL hopefully will improve compared with intensive chemoimmunotherapy [29].

## 5. MCL and Skin Involvement

MCL involving skin is in general a rare event (1%), although the skin represents a site frequently involved in the blastoid variant [23,23,30,31,31,32,33,34,35,36,37,38,39,40]. The clinical and morphological characteristics of MCL at this site are under described with only three series reporting 10, 37, and 9 cases of MCL with cutaneous involvement [23,39,40]. Most patients have a previous history of MCL, and the cutaneous manifestations are associated with a recurrent and progressive disease. In 30% of patients, the cutaneous lesions are the first sign of the disease, making a correct diagnosis more challenging; however, at staging evaluation, all these patients have systemic disease, whereas skin presentation without systemic MCL is a very rare event [36,37,38,41,42].

In the largest series by Kim et al., among a total of 3611 biopsies of MCL cases, only 50 skin biopsy specimens from 37 patients were identified, highlighting the rarity of cutaneous involvement in MCL [23].

**Figure 1:**
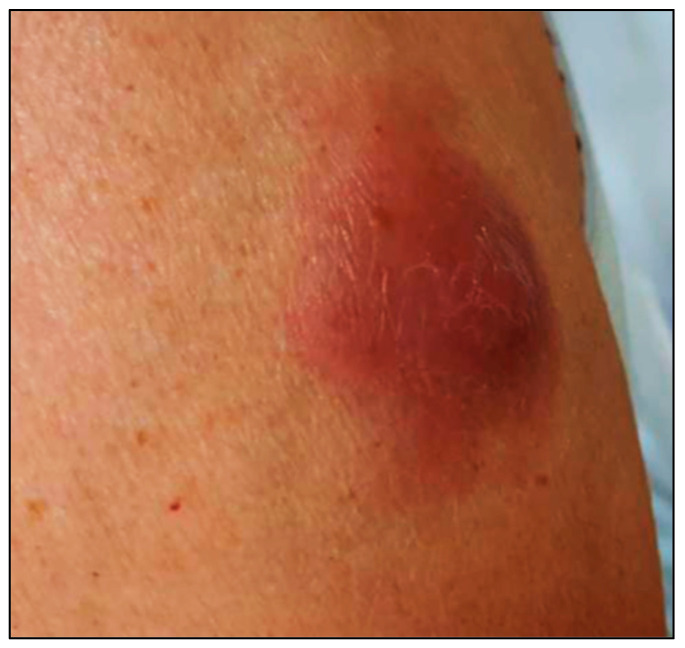
Clinical picture of blastoid MCL presenting with a solitary erythematous nodule on the leg.

The median age of patients at the time of skin disease was 66 years (ranging from 36 to 85 years), with a male predilection (M:F = 2.7:1). Multiple skin lesions were found in 48.6% of patients, whereas 51.4% had a solitary skin lesion. The most commonly involved sites were the extremities (59.3%). Cutaneous lesions were generally nodules or plaques and rarely a disseminated maculopapular rash (Figure 1) [23,40].

The majority of patients (70.3%) presented skin lesions as progression or relapse of a previously diagnosed MCL with cutaneous involvement despite treatment for systemic MCL. A minority of patients (29.7%) presented with skin lesions as the first manifestation of MCL; in this latter group, cutaneous localization was generally a manifestation of a systemic disease and 90.9% of these cases had advanced stage of disease. In the series by Kim et al., only one patient had cutaneous MCL with no other sites involved [23]. 

In the series by Kim et al., multiple lesions were more frequent (81.8%) in patients with skin manifestation as the first sign of the disease, whereas a solitary cutaneous lesion was more common (65.4%) in the group with cutaneous manifestations as disease progression or relapse [23].

**Figure 2:**
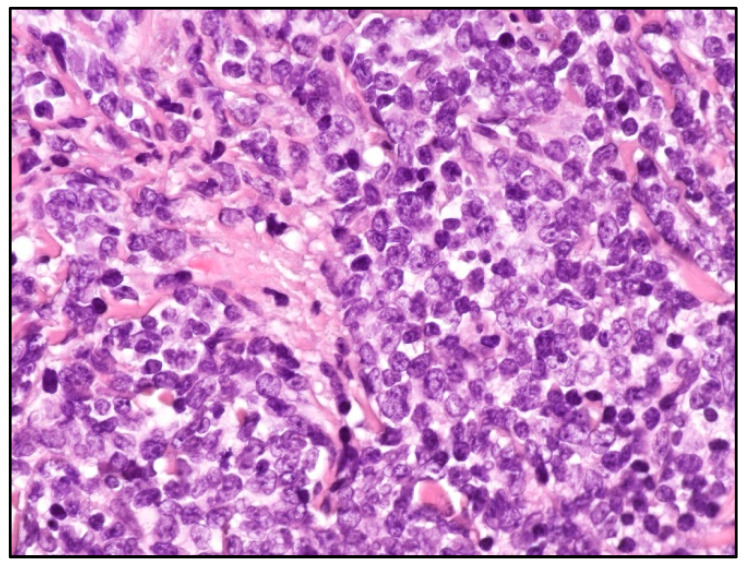
Blastoid MCL: high-power view highlighting cytological features of the disease (Hematoxylin and eosin; magnification 400×).

Histologically, skin involvement in MCL more frequently shows a diffuse pattern of growth within the superficial and deep dermis with or without subcutaneous involvement and sparing of epidermis with a Grenz zone (Figure 2).

**Figure 3:**
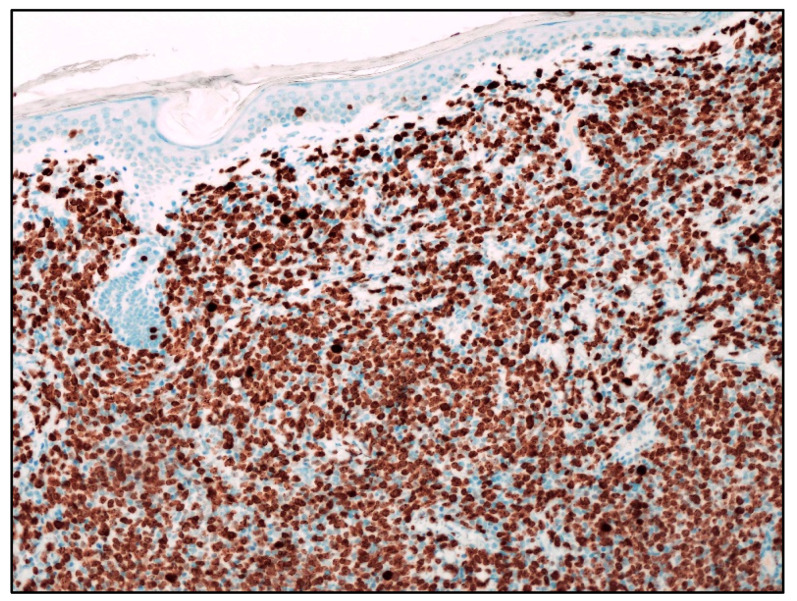
Blastoid MCL: Ki67 immunostaining highlighting the high proliferative index (Ki67 immunostaining; magnification 200×).

In MCL involving the skin, the aggressive cytological subtypes were more frequent (72.9%) compared with the classic subtype (24.3%) [23]. In particular, in the group characterized by skin lesions as relapse/progression of a well-documented MCL, the aggressive subtypes were much more frequent (80%) than the classic subtype (20%) compared with the group with skin lesions as the first sign of disease in which 63.6% and 36.4% of patients showed aggressive and classic variants, respectively. Morphologic progression to an aggressive variant is rather common in patients with cutaneous lesions as disease progression/relapse and 62.5% of patients having classic MCL at initial diagnosis presented aggressive variants at the time of progression/relapse; no patients initially having an aggressive variant relapsed with a classic form. The immunophenotypic features of MCL involving the skin were similar to MCL at other sites, confirming the more frequent CD5 negativity in cases with aggressive morphology as well as a higher Ki67 proliferation rate (90%) compared with the classic variant (20%) (Figure 3).

**Figure 4:**
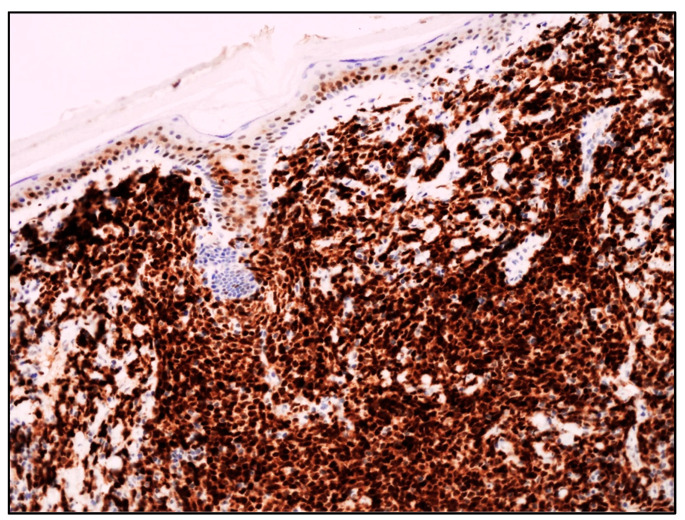
Blastoid MCL: diffuse cyclin D1 expression of neoplastic lymphoid infiltrate infiltrating the dermis (cyclin D1 immunostaining; magnification 200×).

In the series by Kim et al., all cases with skin involvement were cyclin D1-positive and carried *CCND1::IGH* fusion (Figure 4).

Despite chemoimmunotherapy, patients with cutaneous involvement in MCL showed a median overall survival (OS) of 69 months, with no significant difference in OS between patients with skin lesions as initial manifestation of MCL and patients with skin lesions as disease relapse/progression. The prognosis of patients with the aggressive subtypes was worse than that of patients with the classic subtype, with an OS of 59 months vs. 155.8 months. For all patients, the median skin disease to death time was 24.3 months (ranging from 0.4 to 155.8 months). In aggressive MCL variants, the median skin-to-death was 15.9 versus 155.8 months in the classic variant.

## 6. Acute Lymphoblastic Leukemia/Lymphoma (ALL/LBL) of B- or T-Cell Origin

ALL/LBL is a neoplasm derived from precursor lymphoid cells of either B- or T-cell origin [1,2]. The term ALL is used when BM and peripheral blood (PB) are the sites primarily involved, whereas the term LBL is used when lymph nodes or extranodal sites are the primary sites of involvement. 

The majority of ALL is of B-cell origin and occurs in children. The most common type of LBL (90% of cases) is T-LBL and presents with a mediastinal mass with lymphadenopathy. LBL in general affects mainly children and young adults and occurs more frequently in male than in female individuals, with an overall incidence ratio of 2.5:1 [48].

B-LBL is rarer than T-LBL, accounting for approximately 10% of all LBL cases and occurs in children and young adults usually less than 18 years of age. B-LBL frequently involves extranodal sites, such as bone, soft tissue, and skin, or lymph nodes. Mediastinal masses are rarely observed in B-LBL (4%) compared with T-LBL (50–65%). B-LBL has a predilection for cutaneous involvement compared with T-LBL [48,55,57,58].

T-LBL as well as T-ALL affect adolescent males more frequently but may occur at any age. T-LBL frequently presents with a rapidly enlarging mass in the anterior mediastinum, although any lymph nodes or extranodal sites may be involved [1,2].

Although highly aggressive, LBL is potentially curable; overall, B-LBL patients have a relatively favorable outcome compared with T-LBL patients. Despite different disease localization, the therapeutic approach is similar in ALL and LBL, with the main differences concerning B or T cell lineage. Currently, in pediatric patients, the 5-year OS approaches 90%. Modern pediatric programs are based on the intensified use of corticosteroids (mainly dexamethasone), antimetabolites (especially methotrexate and 6-mercaptopurine), and l-asparaginase/pegylated-asparaginase. If minimal residual disease (MRD) is identified, additional dose intensification or allogeneic hematopoietic cell transplantation (allo-HSCT) is planned. Unfortunately, the results in adult ALL/LBL are worse, despite the use of pediatric-type regimens; tyrosine kinase inhibitors in *BCR::ABL1* positive ALL; and bispecific antibodies (blinatumomab), chimeric antigen receptor-T (CART) cells, and inotuzumab ozogamicin in B-cell lineage ALL/LBL, improving the survival to approximately 70% in young adults; however, in older patients, survival decreases progressively to <20%. Nelarabine, a T-cell-specific agent, is used for R/R T-ALL/LBL, with the aim of achieving a response bridging to allogeneic HCT, although the results are variable [81].

## 7. Histology and Immunophenotypic Features of B-LBL and T-LBL

No distinctive morphological characteristics may differentiate B-LBL from T-LBL as in both diseases lymphoblasts are uniform, small- to medium-sized cells with round to oval to convoluted nuclei, fine chromatin, barely visible nucleoli, and scarce basophilic cytoplasm; numerous mitoses are present. At histology, the involved organ is diffusely infiltrated by monomorphic cells, and in case of lymph node involvement, the nodal architecture is diffusely effaced by the neoplastic elements. 

Lymphoblasts of B-cell origin express markers of immaturity (TdT, CD34, and CD10) and B-cell lineage-related markers (PAX5, cCD79alpha, CD19, and CD22). CD20 is expressed relatively late in B-cell differentiation, and B-LBL may lack CD20 expression. The most sensitive and specific B-cell lineage-associated antigen is PAX5; of note, cCD79alpha, PAX5, and CD10 may be found in t(8;21) acute myeloid leukemia (AML), and cCD79alpha may be aberrantly expressed in 10% of T-LBL [45]. MPO may be rarely found in B-LBL, but it may suggest a mixed phenotype only if moderately or intensively expressed.

In T-LBL, the progenitor nature of the cells is demonstrated by the expression of TdT (present in most cases), which may be associated with CD34, CD1a, CD99, and CD117, whereas the T-cell origin is demonstrated by the expression of cytoplasmic CD3 (cCD3), which is the most T-cell lineage specific antigen, as well as surface CD3 (sCD3), which is found less frequently and with a reduced intensity. CD7, CD5, and CD2 expression may be detected [46]. LMO2 in often expressed in T-LBL, with the exception of cases occurring in the setting of myeloid/lymphoid neoplasms with eosinophilia (M/LNs-Eo) [47]. In addition to the aberrant expression of cCD79alpha, markers of myeloid differentiation may be observed in T-LBL. Genetic abnormalities identified in LBL goes beyond the aim of the present paper and are not discussed.

## 8. LBL and Skin Involvement

Although B-LBL is much more uncommon than T-LBL, accounting for only 10% of all LBL cases, skin manifestations are described in 15% to 33% of B-LBL, whereas only 4.3% of T-LBL cases with cutaneous involvement have been reported so far [48,49,50,51,52,53,54,55,56,57,58,59,60,61,62,63,64,65,66,67,68,69,70,71,72,73].

B-LBL is considered the third most common cutaneous lymphoma in children after mycosis fungoides and CD30-positive lymphoproliferative disorders [74].

T-LBL has a male predominance of 2:1, and also, B-LBL was considered to have a male predominance; however, from recent data of the literature, cutaneous B-LBL shows a predilection for young patients (median age 5 years) with a prevalence in females (F:M = 2:1) [48,49,50,51,52,53,54,55,56,57,58,59,60,61,62,63,64,65,66,67,68,69,70,71,72,73]. Cutaneous lesions in B-LBL are more often solitary firm papules or nodules, arising more often in the head and neck area (80%). Awareness of the clinical aspects (a rapidly growing erythematous nodule on the head and neck in young individuals) is essential for a prompt diagnosis and appropriate management [48,49,50,51,52,53,54,55,56,57,58,59,60,61,62,63,64,65,66,67,68,69,70,71,72,73]. The average duration of cutaneous lesions before diagnosis is approximately 3.8 months [48,49,50,51,52,53,54,55,56,57,58,59,60,61,62,63,64,65,66,67,68,69,70,71,72,73].

In B-LBL, skin manifestations are often part of a systemic picture, rather than primary cutaneous disease, being often secondary to BM or lymph node involvement or occurring as sign of disease relapse. As most patients have systemic spread of B-LBL at the time of cutaneous manifestation, a full systemic work-up is essential in patients with cutaneous B-LBL. Cutaneous B-LBL may achieve complete remission and a favorable outcome in more than 70% of cases with B-lineage ALL-type multiagent systemic chemotherapy if therapy is started early.

In cutaneous T-LBL the male to female ratio is 3:1. T-LBL shows a tendency to present as multiple skin lesions (75% of cases), mostly involving the head and neck region (83.3%), although in 41.7% of cases, more than one anatomic area is involved [48,58,75,76,77,78,79,80]. 

Cutaneous involvement in T-LBL is usually a secondary or co-existent manifestation of the disease arising in mediastinum and/or lymph nodes. Recently, our group reported a T-LBL case in which cutaneous lesions were the first sign of the disease and the apparently innocent cutaneous macules were clinically misinterpreted as drug reaction resulting in delay in diagnosis, not being the skin lesions initially biopsied [80].

**Figure 5:**
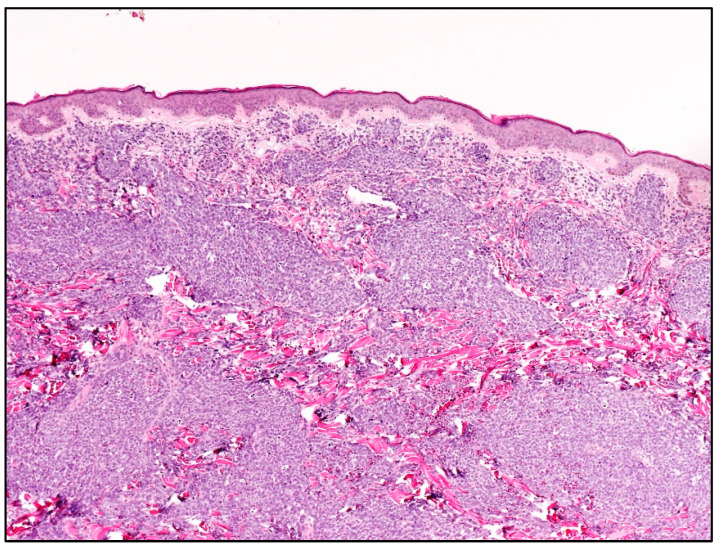
B-LBL: low-power view showing a dense dermal lymphoid infiltrate sparing the epidermis (hematoxylin and eosin staining; magnification 40×).

**Figure 6:**
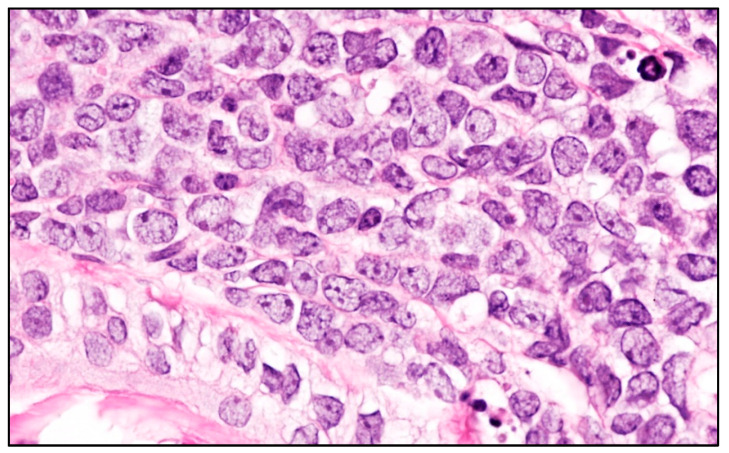
B-LBL: high-power view showing the cytological details of the lymphoid infiltrate with blastic features (hematoxylin and eosin staining; magnification 400×).

Histologically cutaneous involvement by LBL, either of B- or T-cell origin, is characterized by a dense dermal infiltrate of small to medium-sized with cytological features of lymphoblasts lacking epidermotropism and with a Grenz zone beneath the epidermis (Figure 5 and Figure 6). 

**Figure 7:**
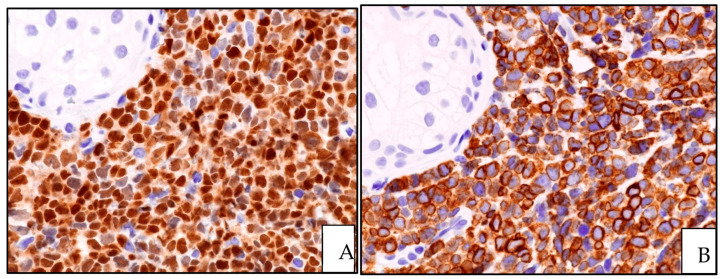
B-LBL: (**A**) TdT positivity of the lymphoid proliferation; (**B**) CD79alpha expression highlighting the B-cell origin of precursor lymphoid cells (TdT and CD79alpha immunostainings; magnification 400×).

**Figure 8:**
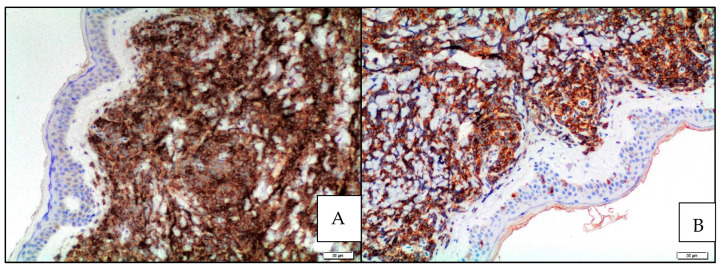
T-LBL: (**A**) CD4 positivity of the lymphoid dermal infiltrate; (**B**) CD1A expression highlighting the precursor nature of the lymphoid infiltrate (CD4 and CD1a immunostainings; magnification 400×).

The neoplastic cells may infiltrate the dermis with a single-file pattern. For the diagnosis of cutaneous involvement in LBL, immunohistochemical precursor markers (TdT, CD34, and CD99) and T- and B-cell antigens (CD2, CD3, CD5, CD19, CD79alpha, and PAX5) should be used to determine whether the tumor cells originated from precursors of B- or T-cell lineages (Figure 7 and Figure 8).

The association of TdT with T-cell marker expression is critical to differentiate skin involvement in T-LBL from the much more frequent event of systemic peripheral T-cell lymphomas secondarily involving the skin. 

LBL is generally regarded as an aggressive lymphoma, although, as previously mentioned, its clinical behavior largely depends on patient’s age, with a worse outcome in elderly individuals [48,53]. Although the number of cutaneous T-LBL cases reported so far is limited, patients with cutaneous B-LBL seem to have a good outcome when treated early with the appropriate systemic multiagent chemotherapy compared with patients with cutaneous T-LBL [48]. In 2020, Bontoux et al. reported the largest case series of ALL/LBL with skin involvement. The authors collected retrospective data from a multicenter cohort of ALL/LBL patients with skin involvement from 13 hospitals from 1997 to 2018. Bontoux et al. reported the clinicopathological features of 38 patients with ALL/LBL with cutaneous involvement, of which 17 were of B-cell origin and 21 were of T-cell origin. Interestingly, the authors noted that cutaneous involvement in ALL/LBL does not seem to be a synonym of dismal outcome by itself. The only characteristics associated with reduced OS were adulthood and relapse during follow-up [82].

## 9. A Focus on Other Hematological Neoplasms Sharing a Blastic Morphology and Presenting Cutaneous Involvement

As already underlined, the blastoid variant of MCL needs to be distinguished from B-LBL and T-LBL, and the positivity for markers such as TdT, CD1a, and CD99, characteristically expressed by lymphoid precursors, generally excludes MCL [43]. Of note, TdT expression has been very rarely reported in aggressive variants of MCL [83,84,85]. The main differential diagnoses are summarized in Table 1. Solid tumors with blastoid features are not discussed in the present paper.

Another hematological neoplasm with a blastic morphology which may involve the skin is DLBCL, in particular primary cutaneous DLBCL, leg-type variant, which is a malignancy occurring mostly on the legs of usually female elderly patients [30,86]. This lymphoma is an aggressive neoplasm with a rather wide spectrum of clinical presentation, more commonly solitary or clustered ulcerated plaques, papules, and tumors. The histological picture is characterized by a dense infiltrate of large cells, mainly centroblasts and immunoblasts, within the dermis and subcutaneous tissue. The neoplastic cells may show angiotropism and epidermotropism and usually express MUM1/IRF4 and sometimes BCL6 with lack of CD10. The presence of cyclin D1 expression in MCL is crucial in distinguishing MCL from DLBCL leg type. 

It needs to be underlined that a diagnostic problem may occur in the small percentage (2.1%) of DLBCL expressing cyclin D1 [87]. The usual lack of CD5 in DLBCL may be helpful in differentiating MCL (generally CD5 positive) from cyclin D1-positive DLBCL; however, it should be reminded that MCL, especially the aggressive morphological variants, may lack CD5 expression [24,25]. SOX11 positivity in MCL is also helpful in the differential diagnosis with cyclin D1-positive DLBCL. The detection of IGH/CCDN1 fusion via FISH analysis confirms the diagnosis of MCL in questionable cases, as cyclin D1-positive DLBCL do not show IGH::CCND1 fusion. In addition, the aberrant expression of CD10 and BCL6, found more frequently in the blastoid and pleomorphic variants of MCL, may prompt an incorrect diagnosis of DLBCL unless cyclin D1 staining or FISH for CCND1 are performed [26]. 

**Figure 9:**
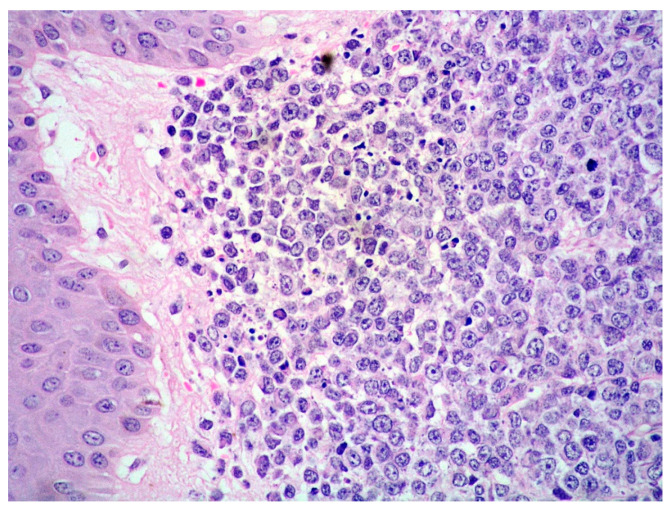
Plasmablastic MM: high-power view showing the cytology of the dermal infiltrate with blastic features (magnification 400×).

**Figure 10:**
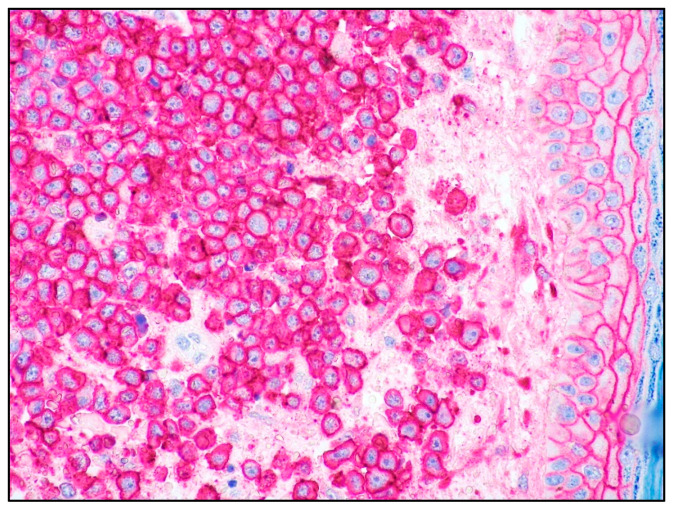
Plasmablastic MM: CD138 expression of the dermal infiltrate supporting the plasma cell origin (CD138 immunostaining; magnification 400×).

Cutaneous involvement is a rare event in multiple myeloma (MM). It may pose diagnostic problems as MM with skin involvement may show a polymorphic or plasmablastic morphology difficult to recognize by morphology alone and leading therefore the pathologists to consider the rather broad spectrum of hematological malignancies with blastoid features. MM is a disease characterized by a clonal proliferation of plasma cells primarily restricted to bone and BM [88,89,90,91,92,93,94,95,96]. The main routes of MM involving the skin are either the hematogenous/lymphatic spread or extension from a contiguous bone lesion. Cutaneous involvement often occurs as a late event in the history of MM, but it may occur at any time in the course of the disease. Skin lesions present as solitary or multiple purple papules or nodules of variable size which may ulcerate. The most frequently involved sites are the chest, lower extremities, back, and buttocks. The histological picture of MM involving the skin is characterized by a nodular; diffuse; or, less often, interstitial proliferation of neoplastic plasma cells involving the deep dermis and subcutis with sparing of papillary dermis and epidermis. The plasma cells may have a mature morphology easily recognizable or a polymorphic and plasmablastic morphology difficult to recognize in the absence of adequate immunostainings such as CD138, CD38, MUM1, and MUM18 (Figure 9 and Figure 10). Of note, MM may show an aberrant expression of T-cell markers, causing diagnostic difficulties [96].

Plasmablastic myeloma needs to be differentiated from plasmablastic lymphoma (PBL), an aggressive lymphoma often associated with Epstein–Barr Virus (EBV) infection, which may rarely involve the skin [97,98,99,100,101,102,103,104,105,106,107,108].

PBL was first described in HIV-positive individuals predominantly in the jaw and oral cavity [1,2,97] and, then, identified in patients with other forms of immunosuppression (IS) such as elderly with age-related immune-senescence, transplanted patients, and individuals with autoimmune diseases, or in the context of iatrogenic IS [98,99]. The disease shows a marked male prevalence, and it occurs at a younger age in HIV-positive patients compared with individuals with other forms of IS. In the setting of HIV-positive patients, PBL is often found to be EBV-positive, as document by EBER. Extranodal sites are predominantly involved in PBL, whereas the skin is rarely affected either in the context of a systemic disease or as a primary site [105,106,107,108]. Cutaneous PBL presents with solitary or multiple grouped and often ulcerated nodules. Histologically, it shows a dermal and subcutaneous infiltrate of atypical cells with plasmablastic, immunoblastic, or anaplastic features; sometimes, plasma cells with more mature features are present. Plasma cell markers (CD138, MUM1) are often positive as well as CD30 and EMA; PBL is often EBER-positive unlike plasmablastic MM. 

**Figure 11:**
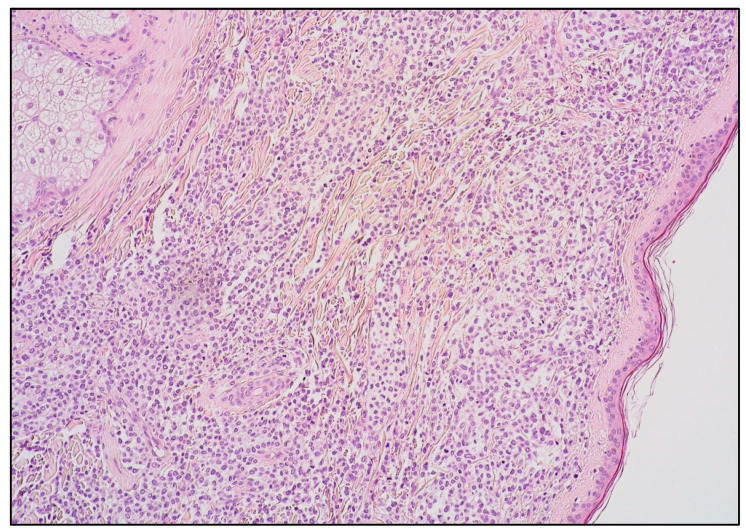
MS: medium-power view showing a dermal infiltrate with single cell filing pattern of growth (hematoxylin and eosin; magnification 200×).

Leukemia cutis is a generic term describing cutaneous involvement in different forms of leukemia. Although any subtype of leukemia may involve the skin, the most common types observed in clinical practice are acute myeloid leukemia (AML) of monocytic or myelomonocytic type and CLL [30,108]. The extramedullary localization of AML is known as myeloid sarcoma (MS) [86,109,110,111,112]. The majority of cutaneous lesions in AML develops in patients with an antecedent diagnosis of leukemia (55–77%). However, cutaneous lesions may appear at the initial presentation of systemic leukemia (23–44%) or even precede the occurrence of leukemia in BM or PB (2–3%). Cutaneous lesions in AML are often firm and rubbery papules or nodules, singular, grouped, or disseminated with no site predilection. Leukemia cutis is considered a systemic manifestation of an underlying leukemia and often follows an aggressive course. In AML without skin involvement, 30% survival at 2 years was observed versus 6% in AML with skin involvement [109]. Most patients with cutaneous manifestations of AML are adults presenting with localized or generalized violaceus papules, plaques, and nodules. Histologically, the cutaneous lesions consist of a dense dermal infiltrate, often extending to subcutis with sparing of the upper papillary dermis and epidermis. The characteristic single cell filing pattern with single files of neoplastic cells between collagen bundles is frequently observed (Figure 11).

**Figure 12:**
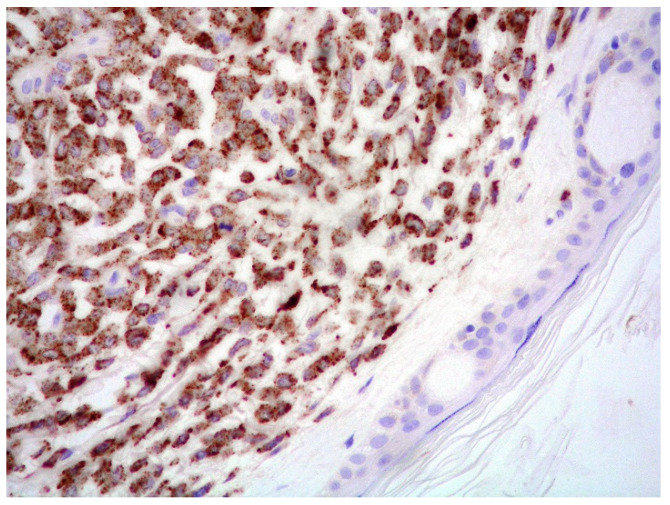
MS: CD68PGM1 positivity of the dermal infiltrate (CD68PGM1 immunostaining; magnification 400×).

The neoplastic cells may show a blastoid morphology in the more immature forms of AML. The phenotype depends on the different subtype of AML, with frequent expression of markers of myeloid differentiation such as MPO and CD15 and markers of monocytic differentiation such as CD163 and CD68PGM1 (Figure 12).

CD43 and lysozyme are often expressed [111,112]. In more immature forms, CD34, CD117 and TdT may be expressed. The frequent positivity of CD4 found in AML together with expression of CD56 in a minority of cases makes the distinction between cutaneous involvement in AML and blastic plasmacytoid dendritic cell neoplasm (BPDCN) very difficult [86].

**Figure 13:**
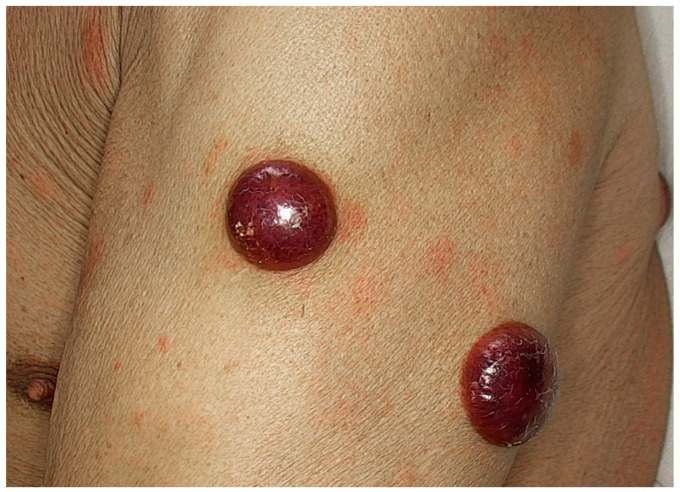
Clinical picture of BPDCN: multiple cutaneous reddish nodules.

BPDCN is an entity that should be included in the differential diagnosis when skin lesions are suspected to be lymphoma/leukemia cutis. BPDCN is an aggressive hematological neoplasm derived from plasmacytoid dendritic cells (PDCs), which may be limited to the skin at presentation [113,114,115]. The skin is the most common site at diagnosis (89%), followed by BM (62%) and lymph nodes (39%). Although skin lesions represent the first manifestation of the disease in the majority of patients (Figure 13), the leukemic spread within a short time (usually between a few weeks or months) from the onset of skin lesions is the rule [9,113,116].

**Figure 14:**
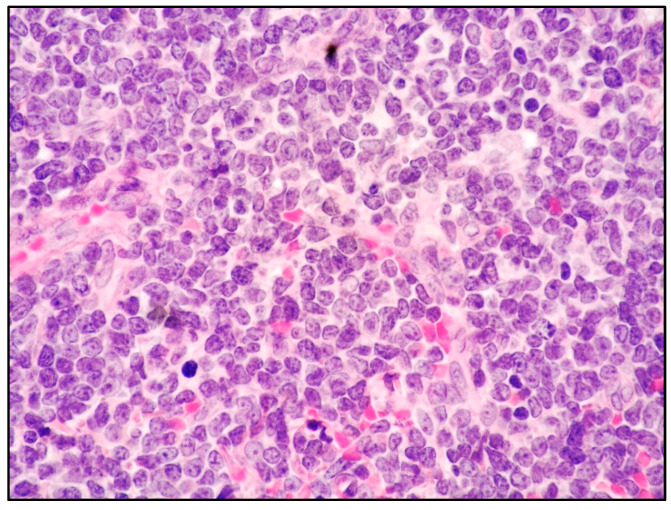
BPDCN: high-power view showing a dense infiltrate with blastic citology (hematoxylin and eosin; magnification 400×).

Unlike LBL, BPDCN is a disease of adults and elderly, whereas pediatric cases are rare and seem to follow a more favorable outcome compared with adult patients [115]. In cutaneous involvement in BPDCN, the dermis and often subcutaneous tissue are diffusely involved due to a monomorphic infiltrate of small to medium-sized cells with a blastoid morphology resembling leukemic infiltration with sparing of the epidermis and Grenz zone (Figure 14).

**Figure 15:**
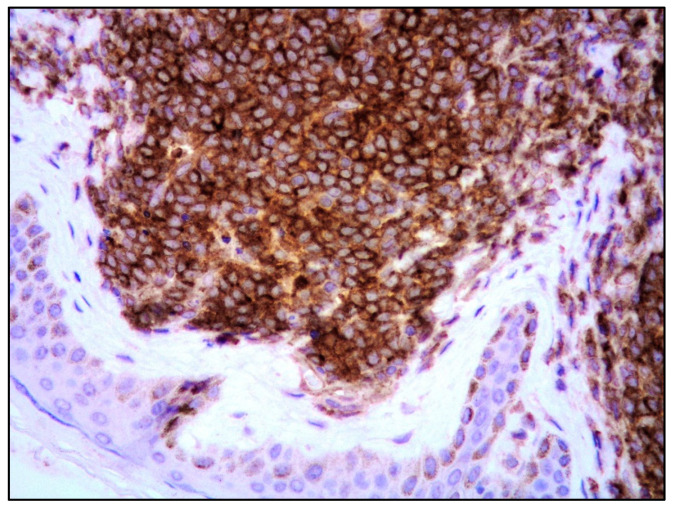
BPDCN: CD123 expression of the dermal infiltrate (CD123 immunostaining; magnification 400×).

A third of BPDCN expresses TdT resulting in misdiagnosing of the disease as LBL. Unlike T-LBL, BPDCN is CD3-negative and usually positive for CD4; CD56; and PDC-associated antigens such as CD123, CD303, TCL1a, TCF4, and CD2AP (Figure 15).

## 10. Conclusions

The differential diagnosis of hematological malignancies with blastic morphology involving the skin includes several entities that, despite similar morphologic features, have different outcomes and require distinct therapeutic strategies. A precise and prompt diagnosis is critical for adequate patient management.

## Figures and Tables

**Table 1:** Clinicopathological features of hematological neoplasms with blastic features involving the skin.

	Sex/Age	Cutaneous Manifestations	Histology	Cell of Origin	IHC	Outcome
**Blastoid MCL**	M:F = 3:1; late sixties	Solitary/multiple papules or nodules; often on extremities. Often previous history of MCL; skin lesions in recurrent and progressive disease	Diffuse dermal and subcutaneous tissue infiltrate; no epidermis and Grenz zone involvement. Monomorphic, medium-sized cells, round/oval nuclei, dispersed chromatin, inconspicuous nucleolus	Mature, naïve B-lymphocytes	CD20+, cyclin D1+, TdT−, CD3−, CD5+ (CD5 loss in 25-28% of blastoid MCL); aberrant expression of CD10, BCL6, CD23; TP53 overexpressed; high Ki67	Poor. Intensive CT and immunotherapy plus ASCT; recently, anti CD19 CART cells, venetoclax, ibrutinib, acalabrutinib
**B-LBL**	F:M = 2:1 children young adults	Often solitary, firm papule or nodule on head and neck. Skin lesions in 15-33% of B-LBL; skin lesions associated with systemic disease	Dense dermal infiltrate; no epidermis and Grenz zone involvement; uniform, small, medium-sized cells, round/oval/convoluted nuclei, fine chromatin, barely visible nucleoli, scarce basophilic cytoplasm; numerous mitoses	Precursor B- lymphocytes	TdT+, CD34+, CD10+, PAX5+, cCD79alpha+, CD19+, CD22+, high Ki67	Good prognosis in 70% of cases with B-lineage ALL-type. Multiagent CT.
**T-LBL**	M:F = 3:1 children young adults	Multiple papules or nodules on head and neck (83.3%); different anatomic areas in 41.7%. Skin lesions rare in T-LBL (4.3% of cases); skin lesions secondary to T-LBL in mediastinum or lymph nodes	Histology identical to B-LBL	Precursor T-lymphocytes	TdT+, CD34+, CD1a+, CD99+, CD117+, cCD3+, sCD3+, CD2+, CD5+, CD7+, aberrant cCD79alpha+, aberrant myeloid markers+	Cutaneous T-LBL are rare, but with a worse outcome compared to cutaneous B-LBL despite multiagent CT.
**MS**	Any age	Localized or generalized violaceus papules, plaques, nodules. Cutaneous MS often in patients with antecedent AML (55–77%) on BM; in 23–44% of cases, cutaneous MS appears at the initial presentation of AML (23–44%) or precedes AML (2–3%).	Dense dermal and subcutaneous tissue infiltrate with sparing of the upper papillary dermis and epidermis. Single cell filing pattern with single files of cells between collagen bundles. Cells with myelomonocytic features or more immature cells with blastic morphology.	Myeloid precursors	Phenotype depends on the subtype of AML (CD34+/−, CD117+/−, TdT+/−, MPO+, CD15+); CD4 and CD56 may be+; CD43 often+; lysozyme often+; NPM1+ in 30% of AML with normal karyotype.	Poor despite intensive CT+ HSCT AML without skin involvement: 30% 2 year survival. AML with skin involvement: 6% 2 year survival.
**BPDCN**	Adults, elderly. Rare in children	Solitary, localized, or generalized brown plaques or nodules. The skin is often the primary site of disease, rapidly followed by leukemic spread.	Diffuse dermal and subcutaneous tissue infiltrate of monomorphic small to medium-sized cells with a blastoid morphology resembling leukemic infiltration with sparing of epidermis and Grenz zone.	PDC	CD4+ CD56+ PDC-associated antigens+ (CD123, CD303, TCL1a, TCF4, CD2AP), TdT+/−, CD3−, MPO−, high Ki67	Poor. CT+ allo-HSCT. High dose MTX+ asparaginase
**DLBCL, leg-type**	F:M = 3:1; often elderly	Solitary or clustered ulcerated plaques, papules, nodules; on legs (>80%)	Dense dermal and subcutaneous tissue infiltrate of large cells (centroblasts, immunoblasts); epidermotropism and angiotropism may be found.	Post-germinal center, mature B-lymphocytes	CD20+, CD79alpha+, Pax5+, TdT−, BCL6+/−, CD10−/+, MUM1/IRF4+, cyclin D1−, high Ki67	Poor (40–50% 5-year survival); anthracycline-based CT+ rituximab
**Plasmablastic MM**	Adults	Solitary or generalized brown plaques or nodules. Skin involvement is often a late event in MM	Nodular; diffuse; or, less often, interstitial proliferation of neoplastic plasma cells with scarce cytoplasm and evident nucleolus, within deep dermis and subcutis with sparing of papillary dermis and epidermis	Plasma cells	CD138+, MUM1+, MUM18+, CD38+, CD20−/+, cyclin D1−/+, CD56+/−, CD79alpha+/− EBER-ISH-, high Ki67	Poor; therapy tailored on systemic manifestations of MM
**PBL**	HIV+ young males or HIV- patients with other causes of IS	Solitary or multiple ulcerated nodules. Skin lesions may occur within a systemic PBL or as primary cutaneous PBL	Dermal and subcutaneous tissue infiltrate of atypical cells with plasmablastic, immunoblastic, or anaplastic features.	Post-germinal center B-lymphocytes	MUM1+, CD138+, EMA+, CD30+, CD20−, EBER-ISH+, HHV8−, high Ki67	Poor despite CT

**Legends**: ALL: acute lymphoblastic leukemia; Allo-HSCT: allogenic hematopoietic stem cell transplantation; AML: acute myeloid leukemia; ASCT: autologous stem cell transplantation; CART: chimeric antigen receptor-T; B-LBL: B-cell lymphoblastic lymphoma; BM: bone marrow; BPDCN: blastic plasmacytoid dendritic cell neoplasm; CT: chemotherapy; DLBCL: diffuse large B-cell lymphoma; EBER-ISH: Epstein–Barr virus-encoded RNA in situ hybridization; HSCT: allogenic hematopoietic stem cell transplantation; IHC: immunohistochemistry; IS: immunosuppression; MCL: mantle cell lymphoma; MM: multiple myeloma; MS: myeloid sarcoma; NPM1: nuclophosmin-1; PBL: plasmablastic lymphoma; PDC: plasmacytoid dendritic cells; RT: radiotherapy; T-LBL: T-cell lymphoblastic lymphoma.
